# On the Origins of Fracture Toughness in Advanced Teleosts: How the Swordfish Sword's Bone Structure and Composition Allow for Slashing under Water to Kill or Stun Prey

**DOI:** 10.1002/advs.201900287

**Published:** 2019-05-02

**Authors:** Felix N. Schmidt, Elizabeth A. Zimmermann, Flynn Walsh, Christine Plumeyer, Eric Schaible, Imke A. K. Fiedler, Petar Milovanovic, Manfred Rößle, Michael Amling, Clément Blanchet, Bernd Gludovatz, Robert O. Ritchie, Björn Busse

**Affiliations:** ^1^ Department of Osteology and Biomechanics University Medical Center Hamburg‐Eppendorf Lottestrasse 55A 22529 Hamburg Germany; ^2^ Materials Sciences Division Lawrence Berkeley National Laboratory Department of Materials Science and Engineering University of California Berkeley CA 94720 USA; ^3^ Advanced Light Source Lawrence Berkeley National Laboratory Berkeley CA 94720 USA; ^4^ European Molecular Biology Laboratory Hamburg Outstation Hamburg 22607 Germany; ^5^ School of Mechanical and Manufacturing Engineering UNSW Sydney NSW 2052 Australia; ^6^ Forum Medical Technology Health Hamburg (FMTHH) Hamburg 22529 Germany

**Keywords:** biomechanical performance, biomimetics, fracture mechanics, toughness

## Abstract

The osseous sword of a swordfish (*Xiphias gladius*) is specialized to incapacitate prey with stunning blows. Considering the sword's growth and maturation pattern, aging from the sword's base to the tip, while missing a mechanosensitive osteocytic network, an in‐depth understanding of its mechanical properties and bone quality is lacking. Microstructural, compositional, and nanomechanical characteristics of the bone along the sword are investigated to reveal structural mechanisms accounting for its exceptional mechanical competence. The degree of mineralization, homogeneity, and particle size increase from the base toward the tip, reflecting aging along its length. Fracture experiments reveal that crack‐growth toughness vastly decreases at the highly and homogeneously mineralized tip, suggesting the importance of aging effects. Initiation toughness, however, is unchanged suggesting that aging effects on this hierarchical level are counteracted by constant mineral/fibril interaction. In conclusion, the sword of the swordfish provides an excellent model reflecting base‐to‐tip‐wise aging of bone, as indicated by increasing mineralization and decreasing crack‐growth toughness toward the tip. The hierarchical, structural, and compositional changes along the sword reflect peculiar prerequisites needed for resisting high mechanical loads. Further studies on advanced teleosts bone tissue may help to unravel structure–function relationships of heavily loaded skeletons lacking mechanosensing cells.

## 1 Introduction

Bone is one of the most remarkable biomaterials found in the animal kingdom, displaying large morphological and functional variability both within and across species.^1^ While the largest loads borne by human bone originate from locomotion, bone structures are also used for hunting or fighting in other species, such as the antlers of deer, the horns of giraffes, or the sword (i.e., rostrum) of the swordfish.^2^ The swordfish (*Xiphias gladius* Linnaeus, 1758) rostrum presents a particularly interesting case as it lacks the osteocyte network that is critical for remodeling of human bone. Thus, it is unknown how the structure of the swordfish rostrum generates and maintains strength and toughness to withstand the large biomechanical forces necessary for hunting.

Bone's resistance to fracture is strongly dependent on the quality of the bone structure, i.e., on the compositional,^3^, ^4^ microstructural,^5^, ^6^ and cellular^7^, ^8^ properties of the tissue. However, the bone quality is characteristically dynamic because bone is continuously resorbed and reformed in the processes of remodeling.^9^ Osteocyte cells, which form a network within the bone, play a critical role in coordinating bone remodeling by sensing stresses and microdamage in bone tissue.^10^, ^11^, ^12^ In this way, changes in the osteocytic network affect the remodeling rate, which can impair bone quality and reduce bone.^7^, ^13^ Swordfish and other neoteleost fish lack osteocytes.^14^, ^15^ Thus, the anosteocytic nature of the rostrum challenges the paradigm that a viable osteocyte network is required to maintain bone quality.^16^ Although devoid of osteocytes, it seems that the sword can still facilitate bone reorganization processes, as reflected by the presence of secondary osteon‐like structures^16^ indicative of bone remodeling.^17^, ^18^ However, if bone remodeling is present in the sword (e.g., to repair damaged tissue), it is likely that the osseous cell behavior, collagen matrix synthesis, and mineralization kinetics differ from mammalian bone remodeling and thus remain to be unraveled in the sword of the swordfish.

The swordfish as a representative of the billfishes uses its eponymous sword to hunt by swiping the rostrum through the water to hit schooling fish. The hit of the sword paralyzes the prey allowing the swordfish to catch and eat the prey. In this way, the rostrum of the swordfish is subjected to high intermittent cyclic bending forces (e.g., the sailfish, another billfish uses its bill experiencing up to 120 ms^−2^)^19^ during hunting. The resistance to these forces originates within the nano‐ and microstructure of the bony rostrum. In bone, the small lengthscales of the mineralized collagen fibrils generate plasticity and the larger lengthscales on the scale of hundreds of micrometers generate toughness by resisting crack initiation and growth.^20^, ^21^ Thus, the ability of the rostrum to resist the biomechanical forces experienced during hunting depends on the integrity of the sword's hierarchical structure and composition.

Additional complexity stems from the fact that the sword grows out from the maxillary bone, which suggests that different lengthwise sites on the sword have different tissue ages,^16^, ^22^ which presumably are related to the sword's length‐related structural and mechanical properties.^16^

Aging and disease affect bone quality^4^, ^7^, ^20^, ^23^, ^24^, ^25^ and can increase the risk of fracture.^26^, ^27^, ^28^, ^29^ Aging effects are strongly associated with reduced osteocyte viability and interconnection,^8^, ^10^, ^13^ bone mineralization,^23^, ^30^ and decreased mechanical competence.^20^ Increasing age, for example, decreases the interface mineralization discrepancy of cement lines and surrounding tissue,^30^ a key factor for toughening.^31^ Lengthwise differences in bone quality can help to reveal the mechanistic material changes that counteract aging effects in bone. Here, we evaluate the origins of fracture resistance in the swordfish rostrum by assessing the quality of the tissue through analyses of the composition and structure as well as the mechanical properties at small and large lengthscales in light of the interaction of the different tissue components. Our objective was to unravel how the bone in the swordfish sword resists high bending forces without suffering from catastrophic fracture.

## 2 Results

### 2.1 Macroscopic Assessment

**Figure 1:**
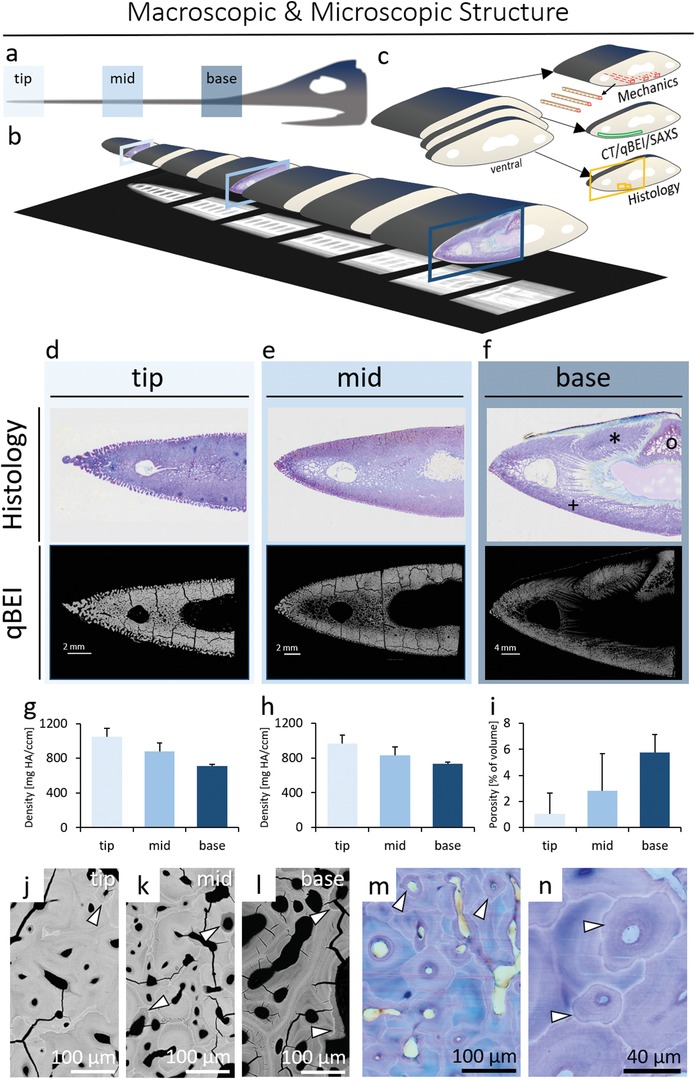
Overview of the rostrum (sword) of the swordfish including cross sections from the tip, middle section, and base. a) Three regions of interest (tip, mid, and base) were identified. b) The sword was cut into seven segments of similar length. At the bottom, X‐rays of the seven segments show a cavity in the center of the sword as well as nutrition canals at the lateral parts of the sword. c) From the tip, mid, and base segments, two consecutive cross sections were cut (thickness of 4 mm). The larger part of the segment (thickness of 90 mm) was used to prepare longitudinal beams for mechanical analyses (red squares). In all cross sections, analysis was performed on the ventral part of the cross section (see color coded squares/ROIs), where a persistent microstructural pattern exists along the sword. One cross section was used for qBEI, SAXS, and CT analysis (green area). One cross section was used for histology (yellow square). Here, the smaller yellow squares reflect in panels (m) and (n). d–f) Toluidine‐blue stained histological sections above quantitative backscattered electron microscopy images (qBEI–quantitative backscattered electron imaging) reflecting mineralization patterns within cross sections. In contrast to d) the tip and e) midsections, f) the base shows the presence of three distinct bony structures, namely the lower premaxillary bone (+), the prenasal bone (*), and the dermethmoid (o).^22^ These bones are fusing during maturation (i.e., already fused in tip and midsections). The brightness of the qBEI measurements reflects the degree of mineralization that is decreasing from d) the tip toward f) the base, while the microstructure becomes denser from the base to the tip. g) HR‐pQCT measurements of mineral density in three swords show overall low interindividual variation in the tip, mid, and base segments, while h) high‐resolution µCT analysis shows the same pattern. However, the bone structure becomes denser from the base to the tip. i) Porosity quantified with µCT shows the same trend. Both density and porosity of 3D µCT‐analysis are strongly correlated with 2D high‐resolution qBEI data (*r*² > 0.98 each for regression of qBEI and µCT). j–l) Quantitative backscattered electron imaging. j) High magnification of a qBEI image of the tip of the swordfish with low porosity. k) High magnification of a qBEI image at the midsection. l) QBEI image with lower mineral content and high porosity at the base. j–l) Arrowheads point to cement lines, which are a sign of earlier resorption and subsequent infilling of newer bone matrix with a lower mineral content. m,n) Histological stainings also reflect the differences in porosity and mineral distribution in the tip section. Of note, no osteocytes are present in the histological cross sections (arrowheads point to secondary osteons pervading primary bone).

The length of the whole sword under study was 0.82 m. The cross section of the tip (distal) had a height of 6.7 mm and a width of 29 mm; the cross section of the base (proximal) had a height of 21.3 mm and a width of 59.8 mm. Contact radiographs acquired in the anterior–posterior direction showed regularly spaced elliptical cavities at the center of the sword (**Figure** **1**, radiographs at the bottom). At the microstructural level, the rostrum cross sections contain a dense outer bony layer forming the cortical‐like shell (Figure 1d–f). Here, osteon‐like structures oriented in the longitudinal direction exhibiting cement line‐like structures were observed. The osteon‐like structures were roughly one order of magnitude smaller than in mammalian bone;^16^ specifically, the inner diameter of the osteon‐like structure, or Haversian‐like canals, was 12.51 ± 8.24 µm at the tip and 28.59 ± 15.63 µm at the base. The surface of the sword was covered by skin (Figure 1f–histology) and displayed a trabecular‐like morphology at the tip (Figure 1d).

### 2.2 High‐Resolution Peripheral Quantitative Computed Tomography (HR‐pQCT) and Microcomputed Tomography

Throughout the length of the swords, i.e., from the base toward the tip, both CT‐based measurements showed a consistently increasing mineralization pattern (Figure 1g,h). Porosity measurements resulted in low porosity at the tip of the sword and a high porosity at the base (Figure 1i), which is consistent with the results gained from quantitative backscattered electron imaging (qBEI) at higher resolution. Regression of qBEI and microcomputed tomography (µCT) data was calculated for both porosity and mineral density with *r*²_porosity_ > 0.98 and *r*²_mineralization_ > 0.98, respectively. The offset of CT‐based porosity values compared to qBEI‐derived porosity (cf. 2.3) originates from partial volume effects associated with CT‐based imaging.

### 2.3 Quantitative Backscattered Electron Imaging

**Figure 2:**
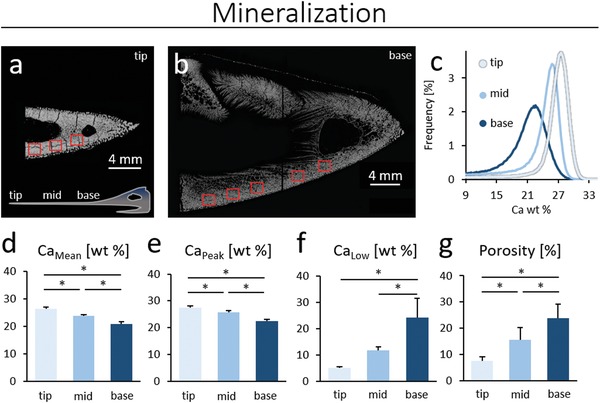
Mineralization patterns in rostral cross sections. Red boxes present regions of qBEI measurements at a) the tip and b) the base. Brighter gray values at a) the tip implies a higher mineralization profile in comparison to b) the base. c) The histogram reveals the bone mineral density distribution given in calcium weight percentages (Ca wt%). d) A significantly declining mean calcium content is measured from the tip to the base. e) Ca_Peak_ shows the same pattern of stepwise declining mineralization values. Ca_Peak_ and Ca_Mean_ values clearly highlight a higher tissue age present at the tip of the sword. f) At the tip of the sword, significantly smaller areas of low mineralized bone are found supporting the notion of the lengthwise aging of bone towards the tip. g) The porosity was derived from qBEI images to account for porosity effects in toughness measurements. A significantly higher porosity can be seen at the base, in comparison to tip and midsections (*p < 0.05).

Quantitative backscattered electron imaging of the rostrum revealed differences in the mineralization distribution for the different cross sections that were examined (**Figure** **2**). The regions of interest (ROIs) examined are shown in the red boxes in Figure 2a,b, with a histogram of the bone mineral density distribution given in Figure 2c for each of these sections. The base of the rostrum was less calcified than the tip.

Specifically, the tip exhibited significantly higher Ca_Mean_ and Ca_Peak_ values as compared to the midsection and base (Figure 2d–e; *p* < 0.05). Furthermore, the tip exhibited a significantly lower Ca_Low_ and lower porosity values than the mid‐section and base regions (Figure 2f,g). The data were normally distributed and analyzed by analysis of variance (ANOVA); the Bonferroni test was applied for post hoc analysis.

### 2.4 Small‐Angle X‐Ray Scattering Analyses

**Figure 3:**
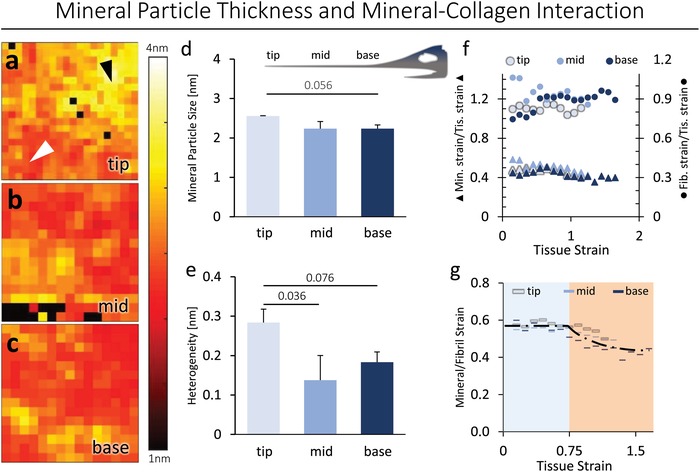
Mineral particle thickness and collagen mineral interaction in the rostrum. a) Mapping of the particle thickness of the tip of the rostrum. The black triangle points to areas with high mineral particle thickness and the white triangle to areas of low mineral particle thickness at the tip. Since the mineral particle thickness differs by a factor of 2 from one area to the other at the tip, a fusion of mineral particles is likely to occur. b,c) The mid and base sections of the sword show mineral particle thicknesses in the same order of magnitude. d) When comparing the three locations, a post hoc test reveals a *p*‐value of 0.056 suggesting intersite differences and lengthwise aging of the bone. e) Particle size heterogeneity in each segment was quantified and a significantly higher variance of particle thickness is found at the tip compared to the mid and base sections. a–c) Each pixel has the size of 50 µm. f) Mineral strain to tissue strain (left axis) and fibril strain to tissue strain (right axis) show no differences along the sword. g) The ratio of the mineral to the fibril strain in the three sections, indicate a constant/elastic region (blue region) and a decreasing mineral strain at the plastic region (orange region).

Maps of the mineral particle thickness measured with small‐angle X‐ray scattering (SAXS) are given in **Figure** **3**a–c. SAXS measurements revealed an average mineral particle thickness of 2.56 ± 0.01 nm at the tip, 2.23 ± 0.18 nm at the midsection, and 2.23 ± 0.09 nm at the base (Figure 3d). The mineral particle thickness varied significantly within each region studied (Figure 3a–c); however, there were no significant differences between regions (Figure 3d), except for a nearly significant difference between the tip and base (*p* = 0.056). However, the heterogeneity of the mineral thickness in the maps per segment was significantly (ANOVA with Bonferroni as post hoc; *p* < 0.05) different between the tip (0.28 ± 0.03 nm) and the mid part (0.13 ± 0.06 nm), while the base (0.18 ± 0.03 nm) showed *p* = 0.076 (Figure 3e).

### 2.5 In Situ Tensile Testing with Real‐Time SAXS/WAXD

Synchrotron SAXS/wide‐angle X‐ray diffraction (WAXD) during uniaxial tensile testing of microbeams of bone from the swordfish rostrum was used to measure deformation of the collagen fibril and mineral during tensile deformation. The mineral and the fibril strains, both as a function of the total tissue strain, did exhibit a constant ratio (Figure 3f). By relating the mineral strain to the fibril strain (Figure 3g), a constant linear region can be seen up to a tissue strain of 0.75%. At higher elongations, the mineral strain to fibril strain decreased, indicating a sliding of mineralized collagen fibrils (fibrillar sliding) with lower elongation in the mineral particles compared to the collagen, thus a sliding between the collagenous and mineral component. Furthermore, the SAXS/WAXD experiments showed a low tissue strain to failure in the tip bone in contrast to a high failure strain in the base bone (Figure 3f).

### 2.6 Fracture Toughness

The fracture toughness of the rostrum was assessed segmentwise in the transverse orientation (with the sharpened starter notch perpendicular to the osteon‐like structures); this is the physiological loading direction of a rostrum bend. The toughness was also assessed in the longitudinal orientation (with the notch parallel to osteon‐like structures) at the base of the sword. The rostrum exhibited a four times higher crack‐initiation toughness and a five to six times higher crack‐growth toughness in the transverse versus longitudinal (nonphysiological) orientations in the same region of the sword (**Table** **1**).

**Table 1:** Toughness specification of the different sword sites: longitudinal specimens did exhibit a far lower resistance to crack initiation and growth. Crack‐initiation toughness values at the tip, midsection, and base did not differ significantly whereas the crack‐growth toughness was highest at the base and lowest at the tip. The error is the standard error of the fitting parameters

Side	Crack‐initiation toughness [MPa √m]	Crack‐growth toughness [MPa √m mm^−1^]
Tip	0.83 ± 0.29	5.17 ± 2.28
Mid	0.88 ± 0.18	9.28 ± 1.54
Base	0.91 ± 0.13	14.8 ± 1.72
Base—longitudinal	0.29 ± 0.20	2.71 ± 1.78

**Figure 4:**
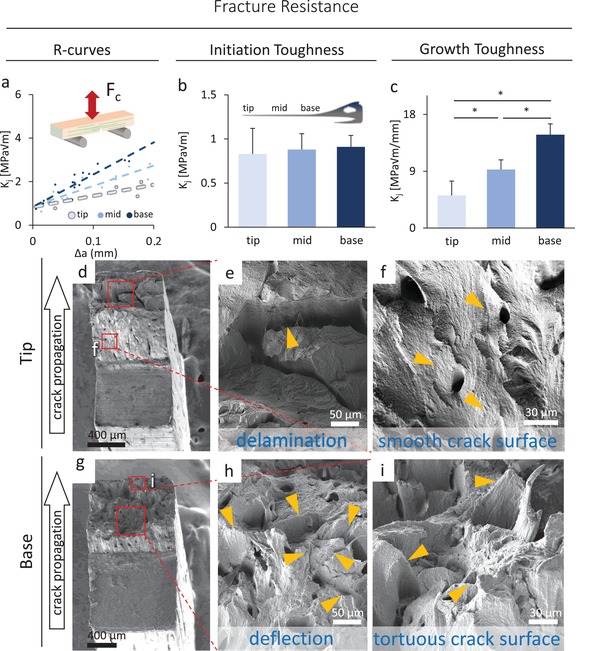
Fracture toughness of the bone material. a) The average (linear fit) *R*‐curves of the specific groups show the material resistance with respect to crack extension. A higher crack‐growth toughness is reflected in a steeper *R*‐curve, as shown by the base material. The inset reflects the experimental setup. b) Crack‐initiation toughness values do not differ between the tested anatomical sites of the sword. Error bars represent the standard error of the fitting parameter. c) Lower crack‐growth toughness was measured at the tip in comparison to the mid and base sections (*p < 0.01 by subset regression analysis, error bars represent the standard error of the fitting parameter). d) An overview of a smooth fracture surface that was found at the tip of the rostrum. e) The crack surface in the swords tip region indicates delamination processes associated with fracture (yellow arrow) resulting in smooth crack surfaces in between the fractured parts, i.e., f) reflecting a brittle‐type fracture, where the present microstructure in the form of small osteon link structures did not affect the path of the crack (yellow arrows). g) An overview of a tortuous fracture surface of a tested specimen from the base region. h) Deflected crack paths reflecting primary extrinsic toughening mechanisms (yellow arrows) are evident at the base. i) The present structural features induce deflection and twisting (yellow arrows) of the crack.

The transverse crack‐initiation toughness was not different between the regions examined (**Figure** **4**a,b); values at the base were only 10% higher than at the tip (Table 1). However, the crack‐growth toughness, a measure of toughness quantifying rising *R*‐curve behavior that is far more sensitive to microstructure,^32^ was significantly greater at the base of the rostrum, roughly two times higher than the midsection and three times higher than the tip (*p* < 0.01) (Table 1, Figure 4c), tested by subset regression. This is visually evident in Figure 4a.

Differences in crack‐growth toughness are reflected in the crack‐surface appearance (Figure 4d–i). Namely, there was mainly delamination at the tip (Figure 4d–f) whereas crack deflection was prominent in the base (Figure 4g–i). Furthermore, the whole crack surface and areas in between delaminated regions (Figure 4f) appeared to be smooth at the tip (Figure 4d,f) and far rougher at the base (Figure 4g,i), the latter being an indication of a more tortuous crack path and, hence, an increased resistance to crack growth at the base.

## 3 Discussion

The bone of the advanced teleost swordfish resists large mechanical loads^19^, ^33^ without fracture despite the fact that it lacks a mechanosensing osteocytic network,^16^, ^22^, ^33^ which is the process used in most damaged bone to orchestrate the remodeling to maintain its material quality. However, signs linked to remodeling have been reported in literature;^16^, ^33^ remodeling has never been explicitly demonstrated in swordfish.^33^ Thus, the swordfish is of particular interest as a model for osteocytic defects and aging. Mechanical examinations of the swordfish bone were previously reported at the macroscale;^22^ however, it is clear that the mechanisms of fracture of the sword (rostrum) and its mechanical competence are reflected more by the swords' peculiar features at the micro‐ and nanoscales. Here, we focus on the microstructural, compositional, and nanomechanical characteristics of the bone material along the sword to understand the micro‐ and nanostructural mechanisms responsible for generating its mechanical integrity in the absence of remodeling–facilitating osteocytes. Our results indicate a predominant influence of the mineral distribution pattern on the crack‐growth toughness behavior of the anosteocytic bone. Furthermore, we report that the rostrum of the sword represents an interesting model of lengthwise aging of bone tissue in a single organism, where an increasing mineral content and mineral homogenization with unchanged fibril–mineral interaction are found at the tip of the sword, as distinct from the base. Our results point to the characteristics of bone suffering from a diminished osteocytic network as in aged human bone,^7^, ^10^, ^34^ or with an absent osteocytic network known from some teleosts.^35^ A low number of viable osteocytes are associated with lower bone turnover and increased mineralization, which result in accumulation of microdamage.^10^

The tip of the rostrum was found to have a significantly higher calcium content compared to the midsection and the base (Figures 1g,h and 2c,d) using high spatial resolution in 2D. These differing mineralization patterns in the tip, mid, and base regions are in line with previously reported results of the rostrum obtained from computed tomography using 0.625 mm resolution.^22^ Moreover, our measurements at the tip reveal a significantly lower content of low mineralized bone (Figure 2f) in line with an increasing homogenization of the mineral distribution toward a narrowing of the mineral density distribution (Figure 2c). Several studies on human bone have shown increasing calcium content with increasing tissue age.^4^, ^30^, ^36^, ^37^ Thus, the mineralization analyses support the hypothesis that the rostrum is older at the tip than at the base.

SAXS analysis revealed a tendency toward higher mineral particle thickness at the tip compared to the base of the sword (Figure 3a–d). Furthermore, there was a clear difference in particle thickness heterogeneity (Figure 3e), where several areas at the tip exhibited a large particle thickness of about 4 nm (Figure 3a). The more heterogeneous and enlarged particle thicknesses at the tip (i.e., at the oldest part of the sword) suggest local fusion of the mineral particles. The observed patterns are compatible with the hypothesized lengthwise aging of human bone, considering that several studies showed an increased mineral particle thickness or size with increased age,^38^, ^39^ as well as a fusion of particles in micropetrosis^8^ or aggregation of grains in aged bone.^40^

To elucidate the influence of the different mineralization level and particle thickness on the collagen–mineral interaction, SAXS/WAXD experiments were conducted along the sword. No differences appeared in fibril strain to tissue strain between all segments (Figure 3f). This is of particular interest because, as some degradation in the collagen quality would be anticipated during aging, a decrease in fibril strain/tissue strain was expected^20^ at the tip of the sword. The latter data imply that the collagen quality is maintained in the rostrum of the swordfish, even at the tip with its higher tissue age. However, no differences in collagen–mineral interaction at the different regions were found (Figure 3g), consistent with in silico experiments that show that an increasing mineral particle thickness does not necessarily have a severe influence on the elastic behavior at lengthscales above 2 nm.^41^

However, comparing our results to those of Gupta et al.,^42^ the bone of the swordfish rostrum exhibits twice as high mineral/tissue strain and fibril/tissue strain as human bone does. This suggests a high degree of plasticity in the matrix and hence higher collagen quality. Also this can be linked to the mineral particle thickness, which was found to be smaller than in the bovine or human bone studied by Gupta et al.^42^ resulting in a nearly doubled fracture strain in swordfish rostrum bone of ≈4%.^22^

Consistent with an unchanged mineral‐to‐fibril strain gradient and unchanged particle thickness, toughness data derived from the fracture‐mechanics experiments revealed a similar crack‐initiation toughness of the sword at the tip, midsection, and base regions. This suggests the differences (Figure 2c–e) in mineralization (by lengthwise aging) do not have a significant influence on the “driving force” to initiate cracking in this bone (Figure 4b).

Clearly, the bone needs to possess an additional ability, not just to withstand crack initiation, but also to resist the subsequent stable propagation of cracks once a crack initiates. The fracture‐toughness experiments here revealed much steeper *R*‐curves for the base material than for the tip, meaning that the material at the base had a higher crack‐growth toughness than at the tip (Figure 4a,c), results which we believe can linked to the nature of the crack path and hence the fracture surface roughness. We observed that the material of the tip of the rostrum displayed somewhat brittle behavior in its fracture mode with smooth crack surfaces and delamination (Figure 4d–f), such that there would have been low energy absorption there,^44^, ^45^ whereas the base exhibited more ductile behavior (Figure 4g–i) which would lead to higher energy dissipation.^5^, ^46^ Specifically, as crack‐growth toughness is invariably associated with extrinsic toughening mechanisms, such as crack‐tip shielding by crack deflection and/or crack bridging, which serve to increase the steepness of the *R*‐curve,^47^ the lower growth toughness at the tip is consistent with the structural features of the bone there rarely acting to deflect the path of the crack (Figure 4d,f) in the aged, and more evenly mineralized, tip material (crack deflection is generally promoted in inhomogeneous bone microstructures; see, for example, Busse et al.^4^). In contrast, the structural features at the base do appear to change the crack path, as shown in Figure 4e–i, and the resulting more deflected crack trajectory is completely consistent with the steeper *R*‐curves (Figure 4a) and 2.8‐fold higher crack‐growth toughness there. Indeed, crack paths in this base region show a degree of twisting in addition to crack deflection (Figure 4i); this is important because crack twisting can be far more potent than in‐plane deflections in enhancing the crack‐growth toughness.^31^

Such different crack extension behavior, where the smother, less deflected, crack paths are associated with more homogeneous microstructures and lower crack‐growth toughness, is a feature of the very different mineralization pattern (Figure 2c), which is not that uncommon in bone, as shown in bone from cases with vitamin‐D deficiency^4^ and Paget's disease.^6^ The bone of the base of the rostrum displayed a very heterogeneous mineral distribution, whereas the tip exhibited a higher and more homogeneous degree of mineralization (Figure 2c). More homogeneous and highly mineralized bone has also been shown to suffer an increased incidence of fracture events in humans.^48^ A lower degree of mineralization with a broad heterogeneous distribution can enable a high extent of crack deflection, and associated deformation, which promotes enhanced crack‐growth resistance. Each interface of low and highly mineralized bone areas can lead to a deflection of the fracture path, primarily from elastic stiffness mismatch across the interface,^46^ which dissipates energy and acts to impede further cracking. A prominent example of this is the mineralization discrepancy of cement line–like structures^16^ to the neighboring osteon‐like structures, which has been shown to be of crucial importance for crack deflection in bone.^31^ An increasing tissue mineralization with age has been shown to accompany a decreasing mineralization discrepancy between osteons and cement lines,^30^ and this is one of the factors associated with the deterioration of the toughness of bone with aging.^20^ We believe that this important effect also is a prime source of the lower (crack‐growth) toughness of the tip of the swordfish's rostrum.

This study though has several limitations. First, we analyzed the rostrum from one individual with the complete experimental array; however, detailed intrarostral differences were quantified in order to link them to compensatory and structural alterations affecting mechanical properties. Therefore, a single animal is sufficient to draw conclusions. In addition, to analyze the effect of interindividual variation, we quantified the mineral density and porosity in the swords of three animals with HR‐pQCT and µCT, and confirmed a consistent pattern of both porosity and mineralization along the sword in all studied rostrums (Figure 1g–i). Furthermore, we did not evaluate full cross sections for SAXS and qBEI measurements; however, representative areas were chosen in each cross‐sectional area, as depicted in Figure 2a,b.

## 4 Conclusions

In conclusion, the sword of the swordfish represents a powerful model of lengthwise aging of bone, as indicated by a mineralization gradient and decreased crack‐growth toughness. Moreover, we additionally demonstrate that a mineral particle size of less than 2 nm had no influence on the ex vivo mechanical properties in our model. We related the gradient in crack‐growth toughness along the rostrum mechanistically to a heterogeneous mineralization pattern, which highlights that of mineralization in bone is of crucial importance to promoting fracture resistance. Finally, we present an intriguing model of anosteocytic bone to study the capability of bone to maintain material quality at low remodeling rates without a network of mechanosensing osteocytes.

## 5 Experimental Section

*Swordfish*: Bone samples were harvested from the rostrum of a *X. gladius*. The fish had a lower jaw‐to‐fork length (LJFL) of 1.95 m and based on this length, the age was determined to be 6–7 years old.^49^ The rostrum was kept on ice prior to preparation. Seven cross‐sectional segments from equidistant regions along the rostrum (100 mm apart) were cut (Figure 1b). For the histological analysis, two consecutive sections with a 4 mm thickness (Figure 1c) were prepared from each segment using a diamond saw (Exakt‐Apparatebau, Norderstedt, Germany). The remaining material was immediately frozen at −21 °C for additional mechanical testing. Cross sections were fixed in 3.5% buffered formaldehyde solution for 48 h and transferred to 80% ethanol until further processing. The tip of the sword, middle segment, and base segment of the rostrum (Figure 1a,b) were used for the bone quality assessment. Two additional swords of swordfish with LJFL of 148 and 159 cm were studied with microcomputed tomography only, to confirm intra‐ and interindividual differences in terms of mineral density and porosity patterns. The study entails no live animals use. The experiments were conducted in line with the Lawrence Berkeley National Laboratory Biological Use Authorization, BUA‐120.

*Contact Radiography*: Contact radiographs of all segments and the appropriate cross sections of the segments were taken for an overview of the structure at 40 kV and an exposure time of 10 s using a digital radiography device (Faxitron, Tucson, AZ, USA).

*Bright Field Microscopy*: For each tip, mid, and base segment, a cross section was embedded in a methylmethacrylate‐based resin (Heraeus Kulzer, Technovit 7200 VLC, Wehrheim im Taunus, Germany) and prepared as ground tissue specimens.^50^ Co‐planar thin sections were ground to a 100 µm thickness using a grinding machine (Exakt Apparatebau, Norderstedt, Germany). Subsequently, specimens were stained with toluidine blue and imaged using an Olympus BX61 microscope (Olympus, Shinjuko, Tokyo, Japan) with a stitched overview at 50‐fold magnification.

*Microcomputed Tomography Analysis*: High‐resolution peripheral quantitative computed tomography (XtremeCT, Scanco Medical AG, Brüttisellen, Switzerland) was conducted in three swords to analyze interindividual variations. Tip, mid, and base segments were imaged using 41 µm voxel size at a tube voltage of 59.4 keV and tube current of 900 µA at a length of 9 mm longitudinally, each. Integration time was set to 200 ms. Mineral density analysis was performed from the center to lateral, part of the ventral rostrum (Figure 1b), mineral density was evaluated using hydroxyapatite phantoms (densities of 99, 204, 406, and 796 mgHA ccm^−1^).

Microcomputed tomography (MicroCT 42, Scanco Medical AG, Brüttisellen, Switzerland) analysis was performed at a resolution of 15 µm voxel size at a tube voltage of 55 keV and tube current of 145 µA on all swords. Integration time was set to 200 ms. Here, porosity was determined at the ventral midpart of the outer shell of the rostrum (Figure 1b). Thresholding was applied to distinguish between bone (371.2–2073.5 mg HA cm^−3^) and soft tissue/voids. Therefore, the ratio of mineralized bone volume and tissue volume was calculated to present porosity values.

*Quantitative Backscattered Electron Imaging*: For each segment, with respect to the bright field microscopy, a consecutive, second cross section (Figure 1b) was embedded in poly(methyl methacrylate) (PMMA) for further assessment of the sword segments.^50^ Briefly, samples were dehydrated and infiltrated with PMMA for 4 days. Specimens were co‐planar ground and carbon coated. Scanning electron microscopy (SEM) was performed at 20 kV and 680 ± 1 pA at a constant working distance of 20 mm (BSE Detector, Type 202, K.E. Developments Ltd., Cambridge, UK; LEO 435 VP, LEO Electron Microscopy Ltd., Cambridge, UK). An aluminum and a carbon standard were used for calibration. Multiple regions of interest (tip: *n* = 5, mid: *n* = 7, and base: *n* = 10) per cross section of the sword were imaged at the ventral part of the sword (Figures 1b and 2a,b for ROI location) with respect to the area of mechanical testing (cf. 5.6 and 5.7; Figure 1b). Results were binned by cross sections. Calcium mean (Ca_Mean_), calcium peak (Ca_Peak_), and calcium low (Ca_Low_) were determined according to standard methods^51^ using a customized MATLAB program (The MathWorks, Inc., Natick, MA, USA). The values of Ca_Mean_, Ca_Peak_, and Ca_Low_ were calculated with respect to the tip mineralization characteristics of the sword. Porosity was calculated from the qBEI images, and ROIs were defined manually using ImageJ (ImageJ, 1.49v, National Institutes of Health, USA–imagej.nih.gov/ij/). During this process of ROI definition, regions with cracks were not included, i.e., cracked regions were excluded from the analysis through a circumnavigation procedure. Statistical analysis was performed utilizing ANOVA with Bonferroni test for post hoc analysis.

*SAXS for Mineral Particle Thickness Measurement*: Mineral particle thickness was quantified using SAXS (P12, Petra III, EMBL‐Hamburg, Germany)^52^ according to the methods described by Gourrier et al.^53^ Tests were performed on multiple ROIs per bone section (tip: *n* = 2, mid: *n* = 2, and base: *n* = 3). The exposure time was set to 5 s, the detector distance was at 3 m for a monochromatic wavelength of X‐rays of λ = 1.3987 Å. Sections of the bone material were cut to a thickness of 10 µm and mounted on aluminum carrier plates. The acquired scattering patterns were background corrected and integrated radially. The *T*‐parameter was calculated according to Gourrier et al.^53^ and was used to describe the smallest dimension of a particle. As a measure for heterogeneity, the standard deviation within each map was calculated based on the set of pixels forming a map.

*In Situ Tensile Testing with SAXS/WAXD*: The deformation of the collagen fibrils and mineral particles in the rostrum during mechanical loading was quantified at the nanoscopic level using SAXS/WAXD at the Advanced Light Source synchrotron radiation facilities (beamline 7.3.3 Lawrence Berkeley National Laboratory, Berkeley, CA, USA).^54^ Here, mechanical uniaxial tensile tests were performed on longitudinal sections from the tip (*n* = 4), mid (*n* = 4), and base (*n* = 4).

These tensile tests were performed to measure the mechanical properties of the samples. Simultaneously, fibril and mineral strains were measured through X‐ray scattering because bone's ordered nanolevel structure (i.e., fibril's 67 nm periodicity and mineral's crystal structure) diffracts X‐rays allowing nanoscale deformation to be measured during tensile testing.^20^ Specimens of tip, mid, and base, 15 mm × 1 mm × 250 µm in size, were prepared using a low‐speed diamond saw (Exakt Apparatebau, Norderstedt, Germany) and kept hydrated during testing. Tests were performed in tension (TST350 tensile stage, Linkam Scientific Instruments, Surrey, UK) with a displacement rate of 1 µm s^−1^. In situ SAXS/WAXD data were collected for 0.3 s every 10 s at an X‐ray energy of 10 keV. The sample detector distance was ≈4000 mm for SAXS and 150 mm for WAXD acquisitions.

The analysis software IGOR Pro (Wavemetrics, Portland, OR, USA) and the custom macro NIKA were used to calibrate the image and convert 2D data to 1D.^55^ Then, the first‐order collagen peak and the mineral (002) peak in the 1D SAXS and WAXD datasets, respectively, were fit to detect changes in the average collagen and mineral *d*‐spacing. Tissue strains were measured by imaging the change in spacing of horizontal lines marked on the sample's surface, which were later analyzed using a custom‐programmed image analysis software utilizing the software package Vision Assistant 8.5 (National Instruments, Austin, TX, USA).

*Fracture‐Toughness Testing*: 25 × 10 mm sections excised from the tip (*n* = 5), middle (*n* = 5), and base (*n* = 5) of the sword, as labeled in Figure 1a, were stored frozen and hydrated prior to testing. Rectangular beams were cut in the direction of the long axis of the sword (with a width of *W* ≈ 2 mm, thickness of *B* ≈ 1 mm, and length of *L* ≈ 10 mm) and lightly polished to yield a visually smooth surface. One surface of each sample (*W* × *L*) was prepared for viewing by polishing with SiC paper to a 1200 grit finish. Each sample was then transversely notched with a low‐speed saw across the midpoint of *L* to ≈0.4 *W* depth and razor micronotched to ≈0.5 *W* with a notch root radius of ≈10 µm. Five samples per location were tested in a Gatan MicroTest 150 N capacity stage (Gatan, Abingdon, UK) configured for three‐point bending with an 8 mm loading span within a Hitachi S‐4300SE/N variable‐pressure scanning electron microscope (VP‐SEM) (Hitachi America, Pleasanton, CA, USA) operating at 35 Pa in variable pressure mode. From the recorded load–displacement data and crack length data measured on the surface of each sample, the *K_J_*_c_ fracture toughness (using a *J*‐based nonlinear elastic fracture mechanics to account for plasticity) was determined in general accordance with ASTM Standard E1820.^56^

In this approach, the linear elastic stress intensity, *K*_el_, is first calculated in the conventional manner as

(1) $$K_{\mathrm{el}}=PSf\left(a/W\right)BW^{3/2}$$

where *P* is the applied load, *S* is the major (three‐point) loading span, and *f*(*a/W*) is a geometry‐dependent function provided in ASTM E1820. Crack lengths were determined from VP‐SEM images taken throughout the failure process. This enables the calculation of *J*_el_, the elastic component of the *J*‐integral, from the standard *J–K* (mode *I*) equivalence relationship *K*_el_ = (*E*′*J*_el_)^1/2^, where under plane‐strain conditions *E*′ = *E*/(1 − ν^2^); for the Young's modulus, *E*, 12.2 GPa and for ν were added, the Poisson's ratio, 0.3 was used. *J*_el_ was then added to the plastic component of the *J*‐integral, *J*_pl_, which was determined from the plastic area, *A*_pl_, under the load–displacement curve as

(2) $$J_{\mathrm{pl}}=\eta A_{\mathrm{pl}}/B\;b$$

where η = 1.9, and *b* is the uncracked ligament width, equal to (*W* – *a*). Finally, *K_J_* was backcalculated from the total *J* (=*J*_el_ + *J*_pl_) using the same standard *J–K* equivalence relationship. For each sample type, these values can then be plotted against the crack extension, Δ*a*, to produce a *K_J_*–*R* (resistance) curve, depicting total toughness and how it changes over the course of failure.

A linear fit was applied to the initial portion of the *R*‐curve for each sample type (Δ*a* < 0.2 mm, which is the limit of validity (*W*/4) as per the ASTM Standard E1820 for the smallest sample tested): nominally, the *y*‐intercept is considered initiation toughness, the material's resistance to crack formation. The slope is crack‐growth toughness, i.e., how the material increasingly resists the propagation of a crack. The indicated uncertainty is the standard error of the fitting parameter.

*Statistics*: Statistical analyses were performed using SPSS 22 (IBM, Armonk, NY, USA). Kolmogorov–Smirnov tests were performed to check the data distribution. In the case of normal distribution, ANOVA and post hoc test (Bonferroni) were used to detect statistically significant differences in structural, histological, and compositional data between the individual segments of the sword. In case of non‐normal distribution, the Kruskal–Wallis test was calculated with Dunn–Bonferroni as the post hoc test. The significance level of α = 0.05 was taken for statistical calculations. For toughness experiments, subset regression analysis was carried out.

## Conflict of Interest

The authors declare no conflict of interest.
